# COVID-Krise und Kinderrechte: Dogmen pandemiegemäßer Alternativlosigkeit auf dem Prüfstand

**DOI:** 10.1007/s12054-022-00467-2

**Published:** 2022-03-02

**Authors:** Michael Klundt

**Affiliations:** Hochschule Magdeburg-Stendal, Stendal, Deutschland

**Keywords:** Kinderrechte, COVID-19, Corona-Pandemie, Kinderarmut, Kapitalismus, Soziale Polarisierung, Solidarität, Soziale Arbeit

## Abstract

COVID-19 und die Maßnahmen dagegen stellen auch die Soziale Arbeit besonders in punkto Kinderrechte vor neue und alte Herausforderungen. Massive Verletzungen der UN-Kinderrechtskonvention und ein Anstieg von Armut in kindlichen und jugendlichen Lebenslagen sind zu verzeichnen. Die Art und Weise der Pandemiebekämpfung war und ist nicht alternativlos (gewesen). Der Beitrag hebt hervor, dass die Kinderpolitik der Regierung über weite Strecken von Instrumentalisierung und Kapital-Konformismus, statt von Kinder-Gerechtigkeit gekennzeichnet war und ist. Das muss sich völkerrechts- und grundgesetzgemäß dringend ändern, indem der Kindeswohlvorrang sowie die Kinderrechte auf Schutz, Förderung und Beteiligung (endlich wieder) umgesetzt werden.

Kinder waren und sind von allen Altersgruppen am wenigsten durch COVID-19 gefährdet, aber am schärfsten von fast allen Corona-Maßnahmen getroffen (worden). Seit Beginn der Corona-Krise in Deutschland wurden elementare Schutz‑, Fürsorge- und Beteiligungsrechte von Kindern und Jugendlichen verletzt und verschiedene (unterlassene) Regierungsmaßnahmen haben überdies zur Verstärkung von Kinderarmut beigetragen.

Das Kindeswohl wurde während der Pandemie durch Entscheidungen der politisch Verantwortlichen nicht vorrangig berücksichtigt, wie in der seit 1992 als Bundesgesetz gültigen UN-Kinderechtskonvention vorgeschrieben (Art. 3). Viele Studien und Stellungnahmen beweisen, dass eine – politisch mit zu verantwortende, strukturelle – Kindeswohlgefährdung im Sinne der UN-Kinderrechtskonvention und des SGB VIII vorliegt, besonders im psycho-sozialen Bereich. Angesichts dessen muss an die Möglichkeit und die Notwendigkeit von Alternativen und Gegenstrategien erinnert werden, die sich am Kindeswohl und an Kinderperspektiven pandemiegemäß orientieren, aber seit zwei Jahren weitgehend politisch, medial und wissenschaftlich ignoriert oder gar stigmatisiert wurden.

Viele Politiker_innen, Journalist_innen und Wissenschaftler_innen vermittelten in den letzten fast zwei Jahren häufig den Eindruck, eine Alternative zu den Corona-Maßnahmen der Bundesregierung würde schlicht bedeuten, dass man viele Corona-Tote zu verantworten hätte (also buchstäblich „über Leichen ginge“, wenn man das Narrativ und die Praxis der Exekutive infrage stelle). Demnach, so einige Vertreter_innen der jeweiligen Professionen, gäbe es gar keine (menschenwürdigen) Alternativen zum Regierungs-Kurs, weshalb sie sich dann auch überwiegend nicht damit beschäftigten. Entgegen vieler öffentlicher Darstellungen formulierte ausgerechnet der Berliner Virologe Christian Drosten jedoch, auf den sich ansonsten die meisten Vertreter_innen der Regierungs-Narrative berufen, bezüglich der Situation im Winter 2021 ziemlich deutliche Widerworte zum hegemonialen Narrativ und zum vorherrschenden Alternativlosigkeits-Dogma: „Es gibt im Moment ein Narrativ, das ich für vollkommen falsch halte: die Pandemie der Ungeimpften. Wir haben keine Pandemie der Ungeimpften, wir haben eine Pandemie.“ Im Interview mit Giovanni di Lorenzo und Andreas Sentker in der ZEIT vom 11. November 2021 sagte Drosten zum ersten Lockdown im Frühjahr 2020 und den Verantwortlichen dafür: „Wir, also die eingebundenen Wissenschaftler, haben gar nicht gesagt, die Schulen müssen geschlossen werden. Andere Behauptungen sind falsch. (…) Das muss die Diskussionsdynamik dieser Ministerpräsidentenkonferenz gewesen sein, nachdem wir den Raum verlassen hatten. Wer das vorangetrieben hat, weiß ich nicht. Ich war nicht dabei. Ich kann nur deutlich sagen: Das war ein rein politischer Beschluss, das ist nicht von der Wissenschaft so empfohlen worden.“ (ebd., S. 34).

Zum zweiten Lockdown bzw. zum Lockdown gegen die sog. zweite Welle Ende 2020 meint der Virologe: „Die Schulschließungen haben die zweite Welle gestoppt. Die Schulen waren das Zünglein an der Waage.“ Hier wäre das französische Gegenbeispiel ein interessantes Diskussionsthema, denn im Unterschied zur BRD waren in Frankreich mit anderer Prioritätensetzung im Winter 2020/2021 die Bildungseinrichtungen (ähnlich wie in der Schweiz) weitgehend offen, ohne ein größeres Infektionsgeschehen aufzuweisen als in Deutschland (vgl. Klundt 2022, S. 105f.). Aber auf die Frage der ZEIT-Journalisten, ob es auch da Alternativen gegeben habe, antwortet Drosten erstaunlicherweise: „Man hätte auch sagen können, die Schulen bleiben offen, aber wir setzen richtig harte Homeoffice-Kriterien im Dienstleistungsbereich durch. Wir nehmen die Wirtschaft in die Pflicht, nicht die Schulen.“ (ebd.) Schließlich beantwortet er die Frage, ob er eine „Abwägung zwischen dem Infektionsrisiko und den Schäden von Kindern im Lockdown“ für legitim hält, mit einem klaren: „Natürlich.“ (ebd.) Daraufhin versetzt er zwar, warum ersteres für ihn gravierender sei, doch die Legitimität einer Abwägung stellt er – im Unterschied zu vielen politischen, publizistischen und wissenschaftlichen Repräsentant_innen – nicht in Frage. Seine Argumentation darf demnach sogar so verstanden werden, dass es bedenkenswerte Alternativen zur Vorgehensweise der Bundesregierung und der Landesregierungen gab und gibt. Und so kann seine virologische Expertise auch zum Teil sozial(arbeits)wissenschaftlicher und kinderpolitikwissenschaftlicher Analysen werden.

## Vergessene Kinder?

Die Wahrnehmung und Interpretation äußerst problematischer politischer Pandemie-Vorgehensweisen gegenüber Kindern scheint sich Schritt für Schritt zu verbreiten. Peter Hanack schreibt im Leitartikel der Frankfurter Rundschau vom 6. Januar 2022: „Schulen bilden die Grundlage für das Funktionieren unserer Gesellschaft. Sie müssen deshalb offen bleiben.“ (Hanack 2022, S. 11) Allerdings lässt er unerwähnt, dass es sich beim (übrigens auch außerschulischen) Recht auf Bildung um ein Kinderrecht und ein Menschenrecht handelt – unabhängig von der Funktionsfrage. Zwar bemerkt er, dass erneute „flächendeckende Schulschließungen (…) ein Verbrechen an der jungen Generation (wären) und (…) unsere Gesellschaft als Ganzes, ihre demokratischen Grundlagen ebenso wie den gesellschaftlichen Wohlstand und Zusammenhalt (gefährden)“, doch die notwendigen Hinweise auf Partizipationsrechte von Kindern und Jugendlichen sowie Beteiligungsrechte von Schülerinnen und Schülern bleibt er weitgehend schuldig. Es hat sich inzwischen herumgesprochen: „Kinder und Jugendliche haben unter der Corona-Pandemie besonders gelitten“, so ein weiterer Artikel in der Frankfurter Rundschau vom Januar 2022. „Sei das nun aufgrund geschlossener Kitas oder Schulen, der Einschränkungen in der Jugendarbeit, im Sport, bei nahezu allen Freizeitaktivitäten. Studien belegen eine Zunahme der psychosozialen Probleme und von Bewegungsarmut bei gleichzeitigem Anstieg der digitalen Kommunikation. Stress, Einsamkeit und Zukunftsangst sind einige der Folgen. Schon ist von der ‚ausgebremsten‘ Generation die Rede.“ (Frankfurter Rundschau v. 06.01.^2022^, S. F3) Meistens wird dabei nicht zwischen der Pandemie selbst und den Maßnahmen dagegen differenziert. Der FR-Journalist Tobias Peter notiert wiederum in einem Kommentar zur Lage der Kinderrechte in Deutschland: „Es sagt etwas über den Charakter eines Landes aus, wie es mit den Kindern umgeht. Nach zwei Jahren Corona zeigt sich: In Deutschland werden bei wichtigen politischen Entscheidungen die Interessen von Kindern und Jugendlichen oft höchstens am Rande bedacht – wenn überhaupt.“ (Peter 2022, S. 27).

## Verleugnete Corona-Maßnahme-Folgen bei Kindern?

Am 7. Januar 2022 berichtete die Berliner Zeitung: „Bis zu 500 Kinder mussten nach Suizidversuchen zwischen März und Ende Mai 2021 bundesweit auf Intensivstationen behandelt werden. Das ist das Ergebnis einer Studie der Essener Uniklinik, über die der Leiter der dortigen Kinder-Intensivstation, Professor Christian Dohna-Schwake, exklusiv im Medizin-Videocast ‚19 – die Chefvisite‘ berichtete. Die Fallzahl sei damit im zweiten Lockdown um rund 400%  im Vergleich mit der Zeit vor Corona gestiegen.“ (Berliner Zeitung v. 07.01.^2022^) Unter anderem als Antwort auf diese Studie, die einen Anstieg von Suizidversuchen unter Kindern im Lockdown feststellte, hat der Bundesgesundheitsminister Karl Lauterbach im Januar 2022 bezweifelt, dass es einen direkten Zusammenhang zwischen dem Lockdown und psychischen Folgen geben könne (vgl. Tagesschau.de v. 11.01.^2022^). In der ARD-Sendung „Hart aber fair“ vom 10. Januar 2022 wies er den Vorwurf zurück, dass die deutsche Corona-Politik mit ihren international vergleichsweise strengen Maßnahmen für die Zunahme psychischer Störungen im Land – vor allem bei Kindern – verantwortlich sei. Er sagte: „Da muss man vorsichtig sein, das geben die Studien aus meiner Sicht nicht her“. Seiner Meinung nach gebe es mehr psychische Störungen auch in Staaten, die weniger gemacht haben als Deutschland. Als Beispiel nannte er die USA, wo seiner Ansicht nach sehr viele Tote vermeidbar gewesen wären oder Großbritannien, wo Lauterbach zufolge gegenwärtig viel zu gefährlich agiert würde. Die psychosozialen Störungen in Deutschland seien seines Erachtens eher der allgemeinen Corona-Lage geschuldet, als auf die Regierungs-Maßnahmen zurückzuführen. „Ich glaube, dass ein großer Teil dieser Probleme einfach an der furchtbaren Pandemie liegt, aber dass das nicht einfach dem Lockdown, den wir praktiziert haben, der damals notwendig war, in die Schuhe geschoben werden darf.“ (Benedict 2022; vgl. Metzger 2022). Dieser Umgang mit Kausalitäten und Koinzidenzen ist auch kindheitswissenschaftlich bzw. kinderpolitikwissenschaftlich bemerkenswert. Denn zweifellos gab und gibt es auch in praktisch allen Ländern dieser Welt psychosoziale Folgen der jeweiligen Corona-Krise und des jeweiligen Krisen-Managements – gerade auch bei Kindern und Jugendlichen. Dennoch lässt sich angesichts der Aussagen des Ministers die Frage formulieren, ob Lauterbach die spezifischen Lockdown-Folgen in Deutschland womöglich nicht ausreichend zur Kenntnis nehmen will.

## Psychosoziale Folgen von Corona und Pandemie-Politik

Viele Studien und Stellungnahmen untermauern, dass buchstäblich eine (politisch mit zu verantwortende, strukturelle) Kindeswohlgefährdung im Sinne der UN-Kinderrechtskonvention und des SGB VIII vorliegt. Das hatte psychosoziale Folgen, wie verschiedene Untersuchungen nachweisen können (vgl. Klundt 2022, S. 175ff.). So hätte sich laut einer Studie des Bundesinstituts für Bevölkerungsforschung hochgerechnet „infolge der Pandemie und der damit verbundenen Schulschließungen bei 1,7 Mio. 11- bis 17-Jährigen die gesundheitsbezogene Lebensqualität erheblich verschlechtert.“ (Bujard u. a. 2021, S. 72) Das Bundesinstitut ermittelte ferner „477.000 Jugendliche im Alter von 16 bis 19 Jahren mit Depressivitätssymptomatik“ (ebd.). Derweil sind die psychosozialen Folgen der Corona-Krise nicht übersehbar. „Ein aktueller Bericht des Robert-Koch-Instituts, der mehrere Studien des vergangenen Jahres zusammenfasst, zeigt: Die Häufigkeit von Angstsymptomen unter Kindern und Jugendlichen ist nach dem ersten Lockdown im vergangenen Frühjahr von 15 auf 24 % gestiegen. Den Eindruck einer verminderten Lebensqualität haben mehr als 40 % der Elf- bis 17-Jährigen. Psychische Auffälligkeiten bei 7‑ bis 17-Jährigen sind von 18 % auf etwa 31 % gestiegen.“ (Vorgrimler 2021, S. 2).

Und, wie auch das „Factsheet“ der Bertelsmann-Stiftung zu „Kinderarmut in Deutschland“ von 2020 ein weiteres Mal nachgewiesen hat, sind die Entwicklungen im Bereich Kinderarmut gerade mit der Corona-Krise noch einmal verschärft worden (vgl. Bertelsmann-Stiftung 2020, S. 1). So wachse mehr als jedes fünfte Kind in Deutschland in Armut auf, was immerhin 2,8 Mio. Kinder und Jugendliche unter 18 Jahren betreffe. Die Kinder- und Jugendarmut verharre seit Jahren auf diesem hohen Niveau. Trotz langer guter wirtschaftlicher Entwicklung seien die Zahlen kaum zurückgegangen. Kinderarmut sei seit Jahren ein ungelöstes strukturelles Problem in Deutschland. „Die Corona-Krise wird die Situation für arme Kinder und ihre Familien weiter verschärfen. Es ist mit einem deutlichen Anstieg der Armutszahlen zu rechnen. Aufwachsen in Armut begrenzt, beschämt und bestimmt das Leben von Kindern und Jugendlichen – heute und mit Blick auf ihre Zukunft. Das hat auch für die Gesellschaft erhebliche negative Folgen“ (Bertelsmann-Stiftung 2020, S. 1).

Ferner haben laut Christine M. Freitag, Professorin für Kinder- und Jugendpsychiatrie und -psychotherapie an der Frankfurter Goethe-Universität, Angst- und Essstörungen sowie Depressionen in der Pandemie bei Kindern und Jugendlichen deutlich zugenommen. Der Wissenschaftlerin zufolge, die auch Vorstandsmitglied der Deutschen Gesellschaft für Kinder- und Jugendpsychiatrie, Psychosomatik und Psychotherapie ist, habe sich zudem die Zeit, die Kinder und Jugendliche an Computer und Handy verbringen, deutlich gesteigert, während sich die meisten viel zu wenig bewegten. Sie widerspricht den Thesen des Bundesgesundheitsministers hinsichtlich psychischer Folgen der Lockdown-Politik: „Hier gibt es mittlerweile Vergleichsstudien aus Kanada und Australien, die deutlich zeigen, dass innerhalb der Corona-Pandemie insbesondere Perioden des Lockdowns zu einem Anstieg der genannten Symptome bei Kindern und Jugendlichen führten. Der Wegfall der Alltagsstruktur, von Bewegung und Sozialkontakten mit Gleichaltrigen ist ein klassischer Risikofaktor für Depressionen und Angststörungen; die Zunahme des Konsums sozialer Medien steht wahrscheinlich in Zusammenhang mit den erhöhten Anorexie-Raten.“ (zit. nach: Schmidt 2022).

Dennoch räumt sie ein, dass man Lauterbach insofern Recht geben müsse, „dass eine echte Kausalität kaum festgestellt werden kann. Allein weil die Experimente dafür – eine Gruppe in einen Lockdown zu schicken und andere nicht – ethisch nicht vertretbar wären“ (zit. nach: Metzger 2022) Wiewohl empirisch strenge Kausalitäten zwischen unterschiedlichen Corona- bzw. Lockdown-Maßnahmen und psychosozialen Folgen bei Kindern und Jugendlichen noch einer tiefgreifenderen Erforschung bedürfen, erscheint die ziemlich strikte Abwehrhaltung des Bundesgesundheitsministers Lauterbach besorgniserregend hinsichtlich des Eindrucks seiner dadurch offenbar ausgedrückten Verweigerung eines politischen Verantwortungsgefühls für die gesundheitliche und psychosoziale Lage der jungen Generation in den letzten zwei Jahren, welches über die Bekämpfung von Corona hinausgeht.

## Regierungs-Kenntnisse von Kinderrechtsverletzungen

Da ist der Wissensstand in der Bundesregierung schon weiter. In ihrem Bericht vom 15. September 2021 zu „Gesundheitlichen Auswirkungen auf Kinder und Jugendliche durch Corona“ fasste die interministerielle Arbeitsgruppe (IMA) aus dem Bundesfamilien- und dem Bundesgesundheitsministerium den bisherigen Forschungsstand zu Corona und Kindern zusammen: „Kinder und Jugendliche haben ein geringes Risiko für schwere COVID-19-Krankheitsverläufe und dadurch bedingte Krankenhausaufnahmen. Doch die sozialen Einschränkungen der Pandemie belasten junge Menschen besonders stark – vor allem diejenigen, die bereits vor der Pandemie unter schwierigen Bedingungen aufgewachsen sind.“ (BMFSFJ/BMG 2021, S. 1) Der interministerielle Regierungsreport für das Bundeskabinett stellt damit unter Beweis, dass auch die Bundesregierung prinzipiell in der Lage ist, wenigstens indirekt zu differenzieren zwischen den Folgen von Corona (Krankheitsverläufe) und den Auswirkungen der Regierungsmaßnahmen (soziale Einschränkungen „der Pandemie“). Dass die Pandemie womöglich soziale Einschränkungen notwendig gemacht haben könnte, sollte dabei nicht in Abrede gestellt werden, aber die Pandemie selbst hat die sozialen Einschränkungen für Kinder und Jugendliche nicht betrieben, deren unterscheidbare Art und Weise nicht beschlossen, sondern die Regierung, die Ministerpräsident_innenkonferenz – und ab und zu auch Parlamente.

## Kapital-Konformismus statt Kinder-Gerechtigkeit?

Der individualisierende Ansatz der Wahrnehmung und Verarbeitung gesellschaftlicher Probleme lässt sich auch z. B. an Aussagen der Ex-Bundeskanzlerin Angela Merkel veranschaulichen. So berichtete die Hannoversche „Neue Presse“ vom 27. Mai 2021: „Sie lasse sich nicht anhängen, dass sie Kinder quäle, soll eine gereizte Angela Merkel in einer der endlosen Länderchefrunden gemurrt haben. Und scherzte in einem Gespräch mit 14 Elternteilen: ‚Eigentlich müsste ich zu jedem von Ihnen nach Hause kommen und mich drei Stunden um Ihre Kinder kümmern, damit Sie auch mal Sport machen können oder Zeit für etwas anderes haben‘.“ (Neue Presse v. 27.05.^2021^) In ihrem Missverständnis der Lage und Probleme von vielen Millionen Kindern und Familien wird ihr gar nicht klar, dass es weder darum geht, ihr persönliche Kinderquäl-Gelüste zu unterstellen, noch sie als Haushaltshilfe oder Babysitterin einzustellen. Kaum eine Familie würde darauf wirklich Wert legen. Vielmehr geht es doch um die allgemeine politische Priorisierung und bundesgesetzlich vorgeschriebene Kindeswohlvorrang-Prüfung bei allen politischen Entscheidungen, welche (mindestens) seit Mitte März 2020 ziemlich in den Hintergrund getreten ist. Da reicht es dann auch nicht, nach über einem Jahr in Online-Konferenzen Interesse an Kindern, Jugendlichen, jungen Erwachsenen, Eltern, Armutsbetroffenen, „Tafel“-Verantwortlichen usw. zu bekunden und dann bei den notwendigen Konsequenzen und Maßnahmen höchstens zu kleckern, statt zu klotzen (man vergleiche nur die seit 2020 enorm gestiegenen Lebenshaltungskosten besonders für Einkommensschwache mit der lächerlichen Hartz IV-Erhöhung um drei Euro im Juni 2021). Auch hier gilt die alte Fußball-Weisheit: Entscheidend ist auf dem Platz.

Wenn im Jahr 2020 staatlich geförderte Großunternehmen Zehntausende von Beschäftigten in von der Solidargemeinschaft mitfinanzierte Kurzarbeit schickten, aber zugleich Milliarden an Dividenden an ihre Großaktionäre de facto von den Steuer- bzw. Beitragszahler_innen finanzieren ließen, deutet sich ebenfalls ein neoliberal strukturiertes Muster der Privatisierung von Gewinnen und Sozialisierung von Verlusten an (vgl. Jaspert 2021, S. 2). Wenn dann bis zum 22. März 2021 zwar alle Bildungseinrichtungen zum mindestens zweimaligen Testen pro Woche gebracht werden können, aber bis dahin weder eine ebensolche Testpflicht für die Unternehmen in der Privatwirtschaft, noch eine Verpflichtung zum möglichen Home Office besteht, lässt sich die gleiche Tendenz erkennen. Wo Tests und selbst leicht mögliches Home Office nur per „Selbstverpflichtung“ erbettelt werden, zeigt auch das eine klare neoliberale Orientierung, wonach der Eingriff in bürgerlich-kapitalistische Eigentumsverfügung ein Tabu darstellt, dem gegenüber Infektionsschutz in einer Pandemie vergleichsweise nachrangig zu sein scheint.

Wenn dann noch trotz öffentlicher Finanzierung von privat-gewerblichen Impfstoff-Herstellern durch Haftungsbefreiung und Patentblockaden die Gesundheit und das Leben von Milliarden von (armen) Menschen auf der Erde hinter die Gewinninteressen der Pharmakonzerne zurücktreten, wird auch hierdurch einmal mehr deutlich, was kapital-konforme Corona-Bekämpfung bedeutet (vgl. Hontschik 2022, S. 48).

## Fazit

Die Hauptkritik an der Bundesregierung aus kindheitswissenschaftlicher Sicht sollte daher vor allem auf die verwertungsorientierte Verobjektivierung der Kinder zielen, welche subjektive Bedürfnisse von Kindern und Jugendlichen – auch jenseits von Kita und Schule – nicht erfragt und somit unberücksichtigt lässt, insbesondere diejenigen Interessen der sozial benachteiligten und vulnerablen Kinder. Ferner ist das Versäumnis einer bundesgesetzlich verpflichtenden vorrangigen Kindeswohlprüfung zu bemängeln, die Verweigerung von Kinderrechten wie Partizipation, Bildung und Gesundheit sowie die Verstärkung von Armutsrisiken ohne Kompensation. Erstaunlicherweise gestehen selbst Regierungsdokumente diese Minimal-Schlussfolgerungen mindestens indirekt selbst ein.
